# Retraction: Curcumin-Free Turmeric Exhibits Activity against Human HCT-116 Colon Tumor Xenograft: Comparison with Curcumin and Whole Turmeric

**DOI:** 10.3389/fphar.2018.00400

**Published:** 2018-04-16

**Authors:** Frontiers Editorial Office

Following publication, concerns were raised regarding the scientific validity of the article. An investigation was therefore conducted in accordance with our established procedures. Following provision of raw data by the authors, the Chief Editor concluded that aspects of the paper's conclusions and assertions were not sufficiently supported by the findings from the material provided. Namely that inconsistencies in experimental rigour preclude confirmation of the conclusion of the article.

The retraction of the article was approved by the Chief Editors of Pharmacology and the Editor-in-Chief of Frontiers.

The authors agree to the retraction.

